# How negative sampling shapes the performance of transcription factor binding site prediction models

**DOI:** 10.1093/bioinformatics/btag048

**Published:** 2026-01-27

**Authors:** Natan Tourne, Gaetan De Waele, Vanessa Vermeirssen, Willem Waegeman

**Affiliations:** Department of Data Analysis and Mathematical Modelling, Ghent University, Ghent 9000, Belgium; Department of Data Analysis and Mathematical Modelling, Ghent University, Ghent 9000, Belgium; Lab for Computational Biology, Integromics and Gene Regulation (CBIGR), Cancer Research Institute Ghent (CRIG), Ghent 9000, Belgium; Department of Biomedical Molecular Biology, Ghent University, Ghent 9000, Belgium; Department of Biomolecular Medicine, Ghent University, Ghent 9000, Belgium; Department of Data Analysis and Mathematical Modelling, Ghent University, Ghent 9000, Belgium

## Abstract

**Motivation:**

Transcription factors (TFs) are key players in gene regulation and development, where they activate and repress gene expression through DNA binding. Predicting transcription factor binding sites (TFBSs) has long been an active area of research, with many deep learning methods developed to tackle this problem. These models are often trained on TF ChIP-seq data, which is generally seen as only providing positive samples. The choice of datasets and negative sampling techniques is a critical yet often overlooked aspect of this work.

**Results:**

In this study, we investigate the impact of different negative sampling techniques on TFBS prediction performance. We create high-quality test datasets based on ChIP-seq and ATAC-seq data, where true negatives can be identified as positions that are accessible but not bound by the TF in question. We then train models using various negative sampling techniques, including genomic sampling, shuffling, dinucleotide shuffling, neighborhood sampling, and cell line specific sampling, simulating cases where matching ATAC-seq data is not available. Our results show that, generally, metrics calculated on training datasets give inflated performance scores. Of the tested techniques, genomic sampling of negatives based on similarity to the positives performed by far the best, although still not reaching the performance of baseline models trained on high-quality datasets. Models trained on dinucleotide shuffled negatives performed poorly, despite being a common practice in the field. Our findings highlight the importance of carefully selecting negative sampling techniques for TFBS prediction, as they can significantly impact model performance and the interpretation of results.

**Availability and implementation:**

The code used in this study is available at https://github.com/NatanTourne/TFBS-negatives (DOI: 10.5281/zenodo.18007567).

## 1 Introduction

The phenotypic diversity observed across life on Earth arises not only from differences in protein-coding genes but also from the complexity of gene regulation. Central to this regulation are transcription factors (TFs), which, together with other DNA-binding proteins such as histones and chromatin remodeling complexes, govern when and where genes are transcribed into RNA. TFs are frequently implicated in diseases. A study on human diseases found that 49 of the 144 investigated developmental disorders could be attributed to mutated transcription factors (Boyadjiev and Jabs 2000). Similarly, TFs are overrepresented among known oncogenes (Furney *et al.* 2006, Vaquerizas *et al.* 2009). Given their key role in regulation and development, TFs and their binding behavior have long been an important area of research. One aspect of this research is the characterization of the DNA sequences that TFs bind to, known as transcription factor binding sites (TFBSs).

### 1.1 Predicting transcription factor binding

It is common to summarize TF binding preferences with Position Weight Matrices (PWMs), often visualized as sequence logos (Crooks *et al.* 2004). Databases such as JASPAR (Castro-Mondragon *et al.* 2022) curate thousands of these profiles. Despite their common use, PWMs provide a simplified view of TF-DNA interactions that fails to capture the full complexity. TFs are not abstract entities that bind a single motif, but a diverse group of proteins that interact with DNA in a multitude of ways. For example, the major TF families—basic helix-loop-helix (bHLH), homeodomain, and zinc finger (ZF)—use different binding strategies (Jones 2004, Razin *et al.* 2012, Bürglin and Affolter 2016), as do many smaller families.

Given this complexity, PWM-based methods are limited: they require arbitrary threshold scores (Mehta *et al.* 2011), assume positional independence, and cannot represent variable-length motifs (Mathelier and Wasserman 2013). Over the years, many improvements have been proposed, including Hidden Markov Models (Drawid *et al.* 2009, Mehta *et al.* 2011, Mathelier and Wasserman 2013), incorporation of DNA shape features (Zhou *et al.* 2015), and other machine learning techniques. More recently, deep learning approaches have become the state of the art.

There are two data types that are most commonly used for training these models: TF ChIP-seq data and Protein Binding Microarray (PBM) data. The former is by far most common and identifies binding sites across the genome by immunoprecipitating TF-DNA complexes and sequencing the bound DNA fragments (Lefrançois *et al.* 2010). PBM data, on the other hand, measures in vitro binding affinities using arrays of short synthetic DNA probes (Berger and Bulyk 2009).

Despite its common use, ChIP-seq data has some drawbacks. For instance, most genomic DNA is not available for TF binding due to the chromatin structure. As a result, TFs only bind at most a few percent of their predicted binding positions (Zaret and Carroll 2011). While this has very interesting implications for cell regulation, it also creates a problem for training models to predict TF binding. Typically, the regions recorded using ChIP-seq are taken as the positive positions, while the rest of the genome is considered negative. If the data is taken as is, the negative positions are not necessarily negative because they are not suitable for TF binding but rather because they were not accessible in the experimental condition. This results in many false negative positions. Additionally, there are many more negatives than positives, leading to very imbalanced datasets further complicating training.

### 1.2 The negatives problem

Multiple strategies have been proposed in literature to address the challenge of finding representative negative data. Our work specifically focuses on methods that use ChIP-seq data. PBM-based methods are not taken into account, as they are in the minority and do not suffer the same complications. Generally, we identified the following broad categories of methods for generating negative samples: linked epigenetic data, base resolution, dinucleotide shuffled, and genomic sampling. We also identified two less common approaches: neighborhood sampling and cross-TF sampling. A summary of relevant examples in the literature can be found in Table 1, available as supplementary data at *Bioinformatics* online.

*Epigenetic data.* One way to address the false negative problem is to use epigenetic data like ATAC-seq, which indicates the accessible regions of the genome. This data can be integrated directly as an additional input track for the models (Cazares *et al.* 2022), enabling epigenetics-aware predictions. False negative positions, where the TF would have bound if it were accessible, now become true negatives in this new setting. In effect, these models are trying to answer a different question: *where do TFs bind given a specific epigenetic context?* Alternatively, the epigenetic data can be used to filter out false negative positions from ChIP-seq data (Quang and Xie 2019, Fu *et al.* 2020). While these are good solutions, they are rarely used as matching epigenetic data is not available for most ChIP-seq experiments.

*Base resolution.* Another approach uses base-resolution predictions, where both negative and positive positions are present in the input window (Avsec *et al.* 2021b, Zhang *et al.* 2021). The models are trained to predict which base pairs actually make up the binding site instead of making a single call for the entire input window. Unfortunately, most ChIP-seq experiments have a low resolution, making this approach only suitable for uncommon, but higher resolution, datasets like ChIP-exo.

*Dinucleotide shuffling.* The most prevalent approach is the use of sampled or synthetic negatives, since it can be applied to any ChIP-seq dataset and also solves the data imbalance problem. For example, negatives are often generated by shuffling the positive sequences while keeping the dinucleotide frequencies intact (Alipanahi *et al.* 2015, Blum and Kollmann 2019).

*Genomic sampling.* Alternatively, some methods ignore the potential false negative problem entirely and generate a set of *hard* negatives by looking for genomic sequences with similar properties like size, GC-content, repeat fraction, or dinucleotide content (Shen *et al.* 2018, Zhang *et al.* 2019). This results in harder, but potentially false, negatives. Interestingly, while having representative negatives is an obvious benefit when training the model, a method that samples negatives that look too much like the positives is likely to be selecting the false negative positions.

*Neighborhood sampling.* Some methods sample negatives by taking sequences in the genomic neighborhood of the positives (Zhang *et al.* 2022a, Zhuang *et al.* 2024).

*Cross-TF sampling.* Finally, some methods use the binding sites of other TFs as negatives (Zhou and Troyanskaya 2015, Quang and Xie 2016, Hiranuma *et al.* 2017). While the latter two approaches are also ways of sampling genomic sequences, they deviate enough from the standard approach to be included as separate categories in our analysis.

The prolific use of these sampled and synthetic negatives raises the question whether they are truly representative of real world data, and consequently, whether the many architectural improvements are truly optimizing for a representative metric. For instance, the work by Ghosh *et al.* (2024) trains models to predict TF binding on a cell line level by using dinucleotide shuffled negatives. They report surprisingly high performance across cell lines, even for TFs that did not occur in the training data. However, these results could also be achieved by a model learning to differentiate between real genomic sequences and dinucleotide shuffled sequences, rather than learning the true binding patterns of the TFs. This question holds for all models using dinucleotide shuffled negatives.

In the absence of large-scale datasets with explicit negatives, the use of synthetic and sampled negatives remains both reasonable and widespread. Nonetheless, for such models to gain broader adoption by biologists, the validity of these techniques must be established. In this paper, we investigate a number of different negative sampling techniques and compare and validate their performance. We limit ourselves to methods that rely solely on TF ChIP-seq data, as this situation is the most common and also the most challenging. Consequently, we do not consider methods that use epigenetic data or base-resolution data, as these would require new experimental data to be generated in most cases.

## 2 Materials and methods

In order to validate the performance of the different negative sampling techniques, we selected a limited number of ChIP-seq datasets belonging to cell lines with linked ATAC-seq data to create *High Quality (HQ)* datasets. We use the ATAC-seq data to select positions that are likely true negatives, defined as being both accessible and not bound by the TF in question. Here, we ignore the effect of possible transient interactions not picked up by the ChIP-seq experiments. For each TF-cell-line combination, we also generated training datasets using one of the following techniques: genomic sampling, dinucleotide shuffling, neighboring sequence sampling, and sampling from other TFs in the same cell line. We then train models for each of these training datasets and test the performance on our HQ *ground-truth* test datasets. In effect, we treat the training datasets as if no matching ATAC-seq data is available, and only use the matched ATAC-seq data for validating the true performance of the models. Finally, we also compare the performance of these models to a simple PWM-based approach and to models trained on the HQ data itself, which serve as lower and upper limit baselines, respectively.

### 2.1 High-quality datasets

The high-quality datasets are based on the data used by maxATAC (Cazares *et al.* 2022). Original ENCODE (Luo *et al.* 2020, ENCODE Project Consortium 2012) ChIP-seq experiments were retrieved from https://hgdownload.cse.ucsc.edu/goldenPath/hg19/encodeDCC/wgEncodeAwgTfbsUniform/, while ATAC-seq data was taken directly from maxATAC (Cazares *et al.* 2022). While maxATAC also published matching processed ChIP-seq data, we retrieved the original datasets from ENCODE to ensure that the data is processed in a way that is more in line with the literature. Positives were defined as the 101 bp windows around the highest point of each ChIP peak. Open ATAC-seq regions were considered accessible and unlikely to contain false negatives, ignoring transient interactions not captured by the ChIP-seq experiments. TF-cell line specific High-Quality (HQ) datasets were then constructed where the negatives are 101 bp frames of DNA centered around the middle of the open ATAC-regions if they do not overlap with a positive for that TF.

A selection of the ChIP-seq and ATAC-seq was performed based on matching the cell lines and TFs, excluding TFs with less than 1000 total positives. This resulted in 119 TF-cell line combinations for the following cell lines: GM12878 (34), K562 (35), HepG2 (32), A549 (14), HEK293 (2), IMR90 (1), and PANC-1 (1). The cell lines HEK293, IMR90, and PANC-1 were only used for hyperparameter optimization while the others were used for training and testing.

A detailed overview of the selected TFs and cell lines can be found in Table 2, available as supplementary data at *Bioinformatics* online. More detailed metrics on the number of positives and assumed *true* negatives per TF-cell line combination can be found in Tables 3–6, available as supplementary data at *Bioinformatics* online. As an additional quality control, the overlap between ChIP-seq positives and ATAC-seq accessible regions was calculated and reported. Generally, a high overlap was observed with the majority of TFs having >90% of their positives located in accessible regions according to the ATAC-seq data. In some cases, like for FOXA1, MafK, MafF, SETDB, and JunD, there was a lower overlap of <50%. This is not surprising as these TFs are known pioneer transcription factors that can bind closed chromatin regions (Sekiya *et al.* 2009, Vierbuchen *et al.* 2017, Warrier *et al.* 2022, Moyers *et al.* 2023). Generally, these deviations do not present a problem, as they only indicate that there are additional true negatives to be found in the closed regions and do not invalidate the negatives found in the open regions.

### 2.2 Training datasets

For each TF-cell line combination, balanced datasets were created using the same positives as the HQ datasets, with a fixed set of negatives generated using one of the following techniques: genomic sampling, shuffling, dinucleotide shuffling, neighborhood sampling, and cell line specific sampling. These represent the most common techniques found in the literature with the addition of shuffled and cell line sampled negatives as potential alternatives. Cell line specific sampling is similar to cross-TF sampling, with the added restriction that only TFs within the same cell line are used.

For the *genomic sampling* approach, the gkmSVM (Ghandi *et al.* 2016) package was used to sample sequence from the genome with similar length, GC-content, and repeat fraction as the positives. Sometimes, no matching sequence could be found. As a result, some of these datasets contain fewer negatives. On average a negative was found for >78% of the positives. *Shuffling* was done by simply randomly changing the order of the nucleotides in the positive sequences. Similarly, *dinucleotide shuffling* was performed by shuffling the positive sequences while preserving the dinucleotide frequencies. This was achieved with code modified from the BiasAway package (Khan *et al.* 2021). For *neighbor sampling*, a random 101 bp sample was taken with the center located 101–200 bp upstream or downstream of the center of the positive position. We also created a *cell line* specific sampling approach where the negatives are sampled from the positives of other TFs within the same cell line. This was done by sampling the positives that match most closely in dinucleotide fraction until an equal number of positives and negatives was obtained. We theorize that binding positions of TFs within the same cell line can be used as a proxy for open positions, although potentially hindered by the fact that they are already bound by the other TFs. Finally, we also trained models on the HQ datasets, which serve as an upper limit for the performance of the models.

### 2.3 Model architecture and training

The goal of this work is to compare different data regimes, not to achieve state-of-the-art performance. Consequently, we designed a relatively simple architecture inspired by existing literature. Similarly, hyperparameter tuning was performed only on the holdout cell lines HEK293, IMR90, and PANC-1 to reduce computational load. Our models are based on the Enformer architecture (Avsec *et al.* 2021a), but exclude the transformer layers. Input DNA sequences of 101 bp are one-hot encoded via a fixed embedding layer into a 4-channel representation. This representation passes through a stack of two 1D convolutional blocks with residual connections, attention-based pooling between blocks, following the Enformer design. A global pooling operation then reduces the sequence dimension, and a linear layer transforms the representation into a single output value. An input size of 101 bp and a CNN kernel size of 9 were chosen because they are standard in the literature. Training was performed using the Adam optimizer (Kingma and Ba 2017) with a batch size of 128 and a learning rate of 2e-4. The specific implementation is available in the code repository on GitHub (https://github.com/NatanTourne/TFBS-negatives).

For each dataset, chromosomes were split into three groups for training, validation, and testing, yielding six cross-validation configurations for each TF-cell line combination. Group 1 contained chromosomes 3, 4, 7, 13, 18, 19, 20, and X; group 2 contained 1, 9, 10, 11, 15, 21, 22, and Y; and group 3 contained 2, 5, 6, 8, 12, 14, 16, and 17. For each TF, cell line, and negative type combination, six models were trained—one per cross-validation split. Training proceeded using the training split until overfitting occurred on the validation split. Each model was then evaluated on the test split of both the training dataset and the HQ dataset. Specific details on the number of positives per split for each TF-cell line combination can be found in Tables 3–6, available as supplementary data at *Bioinformatics* online. Full training code is available on GitHub.

Model performance was evaluated using the Area Under the Receiver Operating Characteristic curve (AUROC). The AUROC was calculated for each cross-validation split and averaged across splits for each TF-cell line-dataset combination.

## 3 Results and discussion

### 3.1 General performance

**Figure 1:**
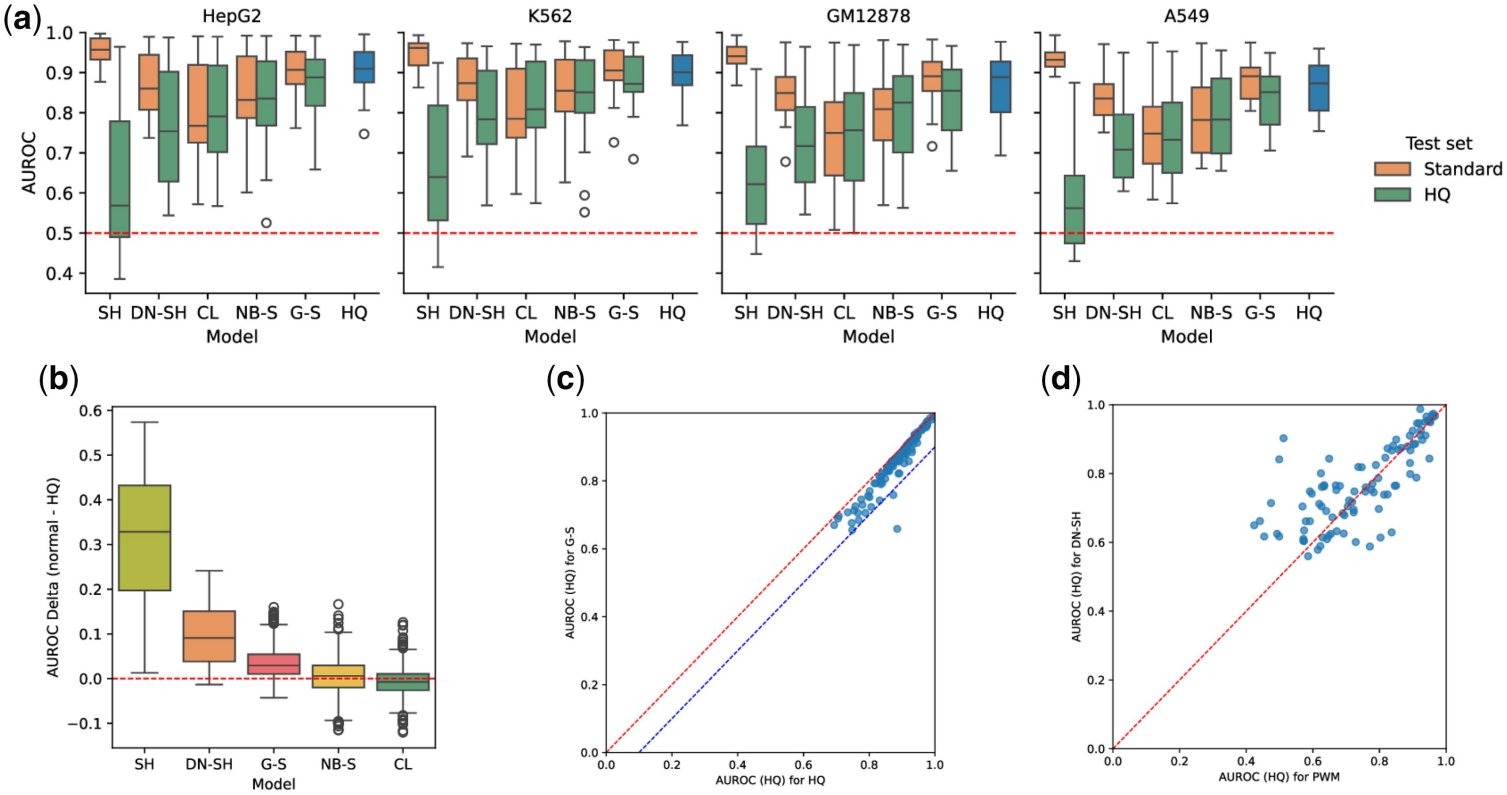
(a) AUROC performance on the test splits of the training and high-quality datasets. The *x*-axis shows dataset types: *SH* (shuffled), *DN-SH* (dinucleotide-shuffled), *CL* (cell line negatives), *NB-S* (neighbor sampling), and *G-S* (genomic sampling). Models trained on HQ datasets (*HQ*) are also shown. The dashed line indicates random performance at 0.5 AUROC. (b) The difference in AUROC scores between training and high-quality datasets. (c) A detailed comparison of the performance of the models trained on high-quality and genomic sampled datasets. The diagonal line represents equal performance. The addittional line indicates 10% better performance on the HQ datasets. (d) A detailed comparison of the performance of the models trained on dinucleotide shuffled datasets and the results of the PWM-based approach. The diagonal line indicates equal performance.

We trained models on each training dataset as described previously and evaluated their performance on the test split of the training and the high-quality (HQ) datasets. The results are shown in Fig. 1a. Performance across cross-validation splits was averaged, so each point represents the mean AUROC for a specific TF-cell line combination. The performance on the training dataset and the high-quality dataset will be referred to as *standard* and *HQ* AUROC, respectively. There is a substantial difference in standard AUROC scores across dataset types. Models trained on shuffled and genome-sampled datasets achieve high standard AUROC scores, with averages above 94% and 89%, respectively, whereas those trained with cell line sampling perform worst, with an average AUROC score of around 77%. However, this likely reflects the difficulty of the task presented by each dataset. For instance, high performance on shuffled datasets indicates that the model is able to learn the difference between real genomic positives and shuffled sequences, which is perhaps an easy task. Whether this corresponds to recognizing binding sites is not proven by these results. Genome-sampled datasets also perform well. Here, negatives are genomic sequences with similar properties, it is more reasonable to assume that the model needs to recognize binding sites in order to perform well. The substantially lower performance on datasets based on neighboring negatives is curious. One would expect this task to be similar or even easier than the genomic sampling task, yet the average standard AUROC score was around 82%. The fact that performance is substantially lower might indicate that there is some binding signal in the neighboring sequences, or that regions with TFBSs have some other characteristics not picked up in the genomic sampling approach. As expected, with an average AUROC around 86%, models trained on dinucleotide-shuffled datasets perform worse than those trained on simple shuffling, since dinucleotide shuffling is a harder task. Surprisingly, their performance is also lower than genome-sampled datasets.

### 3.2 Performance on high-quality datasets

When the same models are evaluated using high-quality datasets the situation changes drastically. On high-quality datasets, models trained on genome-sampled datasets perform the best, with an average HQ AUROC around 86%, followed by other methods based on sampling real genomic sequences, namely neighbor sampling and cell line sampling, with average HQ AUROC scores around 82% and 78%, respectively. Models based on shuffled or dinucleotide shuffled datasets struggle with real-world data, with average HQ AUROC scores around 63% and 76%, respectively. Performance for the simple shuffling approach is especially poor. Under the naive approach, standard AUROC scores often exceeded 90%, yet performance on high-quality datasets is barely above random. This confirms that these models are not learning the true binding patterns of TFs, but rather learning to differentiate between real and shuffled genomic sequences. The fact that dinucleotide shuffled datasets performed worse on standard AUROC but outperformed shuffled datasets on HQ AUROC is a logical consequence. For datasets based on genomic sampling, neighbor sampling, and cell line sampling, performance on HQ datasets more closely matches that on their respective training datasets. This indicates that these datasets are more representative of real-world data and that the models are learning to recognize binding sites. Among these, cell line sampling performs worst, likely because it optimizes towards a slightly different task. The models are trained to reject binding sites of other TFs, a harder task, resulting in lower standard AUROC performance. At the same time, negatives from the HQ datasets are mostly open genomic regions, and not binding sites of other TFs.

These patterns are consistent across cell lines, suggesting the conclusions generalize well. To see if these patterns hold for individual TFs, detailed comparisons are shown in Fig. 1, available as supplementary data at *Bioinformatics* online. For each TF the best performing negative sampling method on the HQ dataset is shown. As expected genomic sampling is the best performing method for the majority of TFs. However, neighborhood and cell line sampling do outperform genomic sampling for some TFs. Out of the 690 different TF-cell line-cross-validation combinations, genomic sampling was the best performing method in 593 case, followed by neighborhood sampling with 85 cases. In some rare instances, cell type negatives and even dinucleotide shuffled negatives were the best performing, with 11 and 1 cases, respectively. From this it is clear that as a general rule genomic sampling is the best choice, but for specific TFs other methods might be more suitable. Interestingly, we see that for CTCF genomic sampling is almost never the best performing method. Similarly, MafK, MafF, JunD, and FOXA1 also show a preference for neighborhood or cell line sampling.

**Figure 2:**
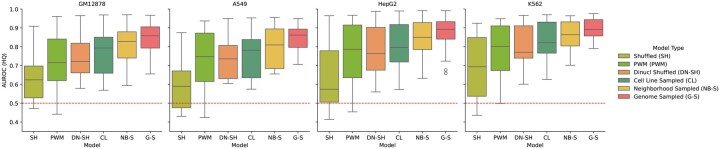
Performance on HQ datasets for different training strategies, including a PWM-based baseline. TFs without available PWMs were excluded across all models used in the figure.

While the models were optimized for AUROC performance, we also calculated the Area Under the Precision-Recall Curve (AUPRC), Accuracy, Matthews Correlation Coefficient (MCC), Precision, and Specificity. To calculate metrics where thresholding is necessary, we have calculated the optimal thresholds based on the validation splits of the standard datasets. The results, shown in Fig. 2, available as supplementary data at *Bioinformatics* online, follow similar trends as the AUROC results. Generally there is a big drop in performance when moving from standard to HQ datasets. This drop is more pronounced for AUPRC, Precision, and MCC, which are more sensitive to class imbalance. The relative performance of the different negative sampling methods on the HQ test set is mostly consistent across metrics. The shuffling based methods perform worst, while the sampling based methods perform best. However, there is some variation in the ranking of the individual methods. While genomic sampling performs best for AUROC and AUPRC, it is actually outperformed by neighbor and cell line sampling for Accuracy, MCC, and Precision, suggesting that different sampling methods might be more suitable depending on the metric of interest. However, it should be noted that all models were optimized for AUROC performance, and different results might be obtained if the models were optimized for other metrics.

High-quality datasets are constructed using matched ATAC-seq data, which is not available for most ChIP-seq experiments, and so this type of verification is not possible for most datasets. Consequently, it is not just important to find a universal method of generating the negatives that achieves high performance on the HQ datasets, but also one that reports AUROC scores on its own datasets that are representative of the true performance. Towards this end, Fig. 1b shows the absolute difference in AUROC scores between training and high-quality datasets. Note that this difference was calculated for each cross-validation split before being averaged for each TF-cell line-dataset combination. Shuffling and dinucleotide shuffling based datasets show the largest difference. Neighbor and cell line based sampling methods show almost no bias. This suggests these methods serve as reliable proxies for HQ datasets. Genomic sampling, the highest-performing method on HQ datasets, does show some limited bias. This might be because the sampling approach results in a dataset that is noticeably different from the HQ one. This unfortunately implies a trade-off between higher performance and accurate performance estimation.

### 3.3 Training on high-quality datasets

The blue boxplots in Fig. 1a shows the results for models trained on the HQ datasets themselves. This represents the best case scenario for the performance of the models, only achievable when ATAC-seq data is available. In general, models trained on the HQ datasets outperform all other models, with an average HQ AUROC score of around 89%. This indicates that the other datasets are still not fully representative of real-world data. Figure 1c shows a detailed comparison between the performance of the models trained on HQ and genome-sampled datasets. In general, genomic sampling achieves AUROC scores that are relatively close to the best case scenario.

### 3.4 PWM-based approach

Despite over a decade of deep learning based methods, the use of PWMs remains common in the field. Consequently, we include a naive PWM-based approach as a worst case baseline. For each TF, we retrieved matching PWMs from JASPAR (Castro-Mondragon *et al.* 2022), as summarized in Table 7, available as supplementary data at *Bioinformatics* online, and used them to scan the input sequences using a basic Biopython (Cock *et al.* 2009) implementation. The highest score over the input window was used as the prediction. If multiple PWMs were found, all were used for scanning and the highest score per input window was used. AUROC scores were calculated on high-quality datasets and the results are shown in Fig. 2. Since PWMs could not be found for all TFs, those TFs were also excluded from the comparison across all models. Surprisingly, the PWM-based approach performed well, consistently outperforming shuffling, and reaching performance on par with dinucleotide shuffling and close to cell line sampling. Only neighborhood and genomic sampling clearly outperformed the PWM-based approach. These results are very surprising, especially since the use of dinucleotide shuffled negatives is so common in the field. To further investigate this, a detailed comparison is shown in Fig. 1d. The results are fairly evenly split between performance being better for the PWM-based approach and for the dinucleotide shuffled based models. The main takeaway here is not the absolute performance, but rather that for a given deep learning architecture, performance varies widely with the choice of negatives. Furthermore, this choice of negatives can be the difference between a model that is an improvement over the capabilities of a simple motif scan and one that is not. Most articles in the literature are focused on ever improving architectures to achieve higher performance. These results show that the choice of negatives is an undervalued part of the story.

### 3.5 Effect of dataset size

**Figure 3:**
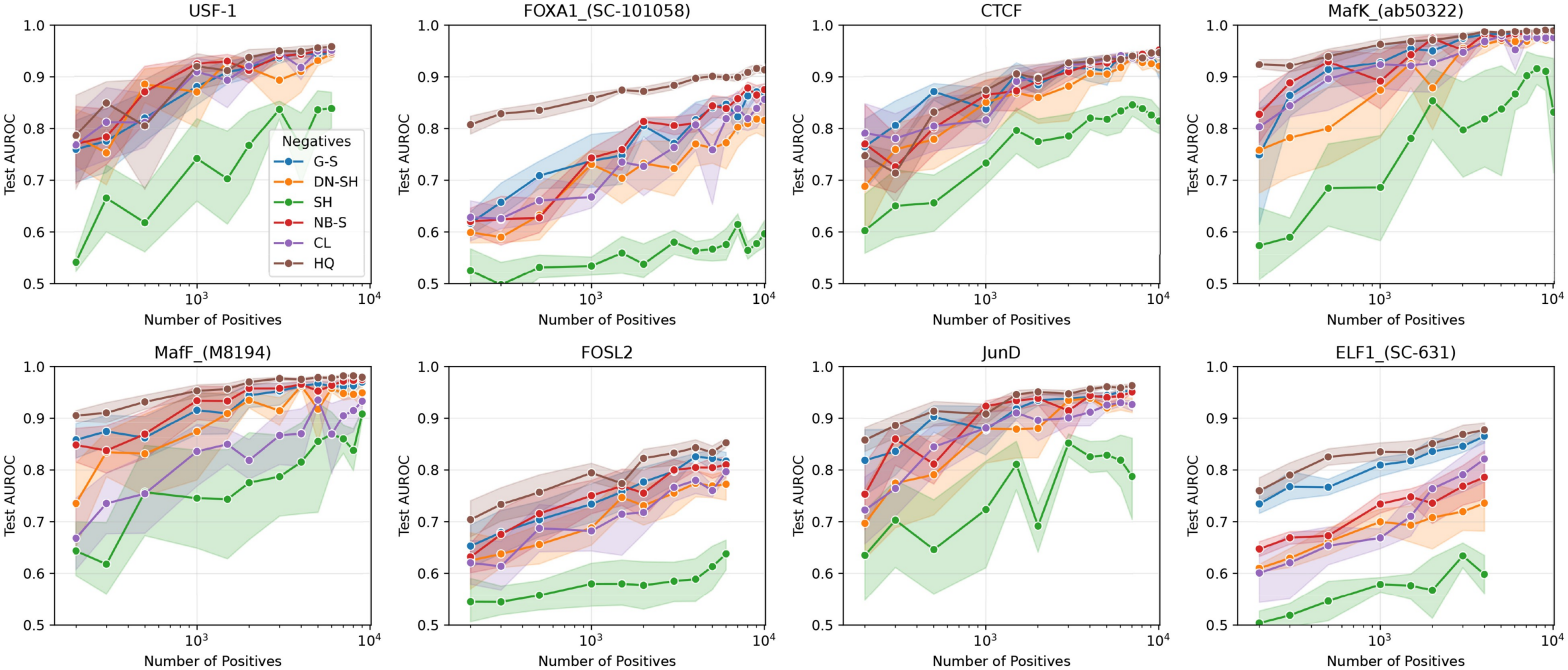
Effect of training positives on HQ performance for eight TFs in HepG2. On the *x*-axis the number of training positives is shown, while the *y*-axis shows the AUROC performance on the HQ test set. Each subplot shows a different TF, indicated on the top. The different colors indicate the different types of negatives used during training. The *x*-axis shows dataset types: *SH* (shuffled), *DN-SH* (dinucleotide-shuffled), *CL* (cell line negatives), *NB-S* (neighbor sampling), and *G-S* (genomic sampling).

We also examined the effect of the number of training positives. For the *HepG2* cell line, we selected eight TFs with training datasets containing large numbers of positives. Models were trained with an artificially limited number of positives: 200, 300, 500, 1000, 1500, and 2000–10 000 in increments of 1000. The performance of these models for each negative type on the high-quality dataset is shown in Fig. 3. There is a clear trend that the performance increases with the number of training positives. However, some TFs, like *USF-1*, *MafF*, *JunD*, and *MafK*, show a plateau, where performance gains diminish as the model approaches its maximum. TFs where the models do not show this plateauing effects are likely not achieving their maximum performance yet, and could benefit from more training positives. Interestingly, for some TFs, there is a large performance difference between negative types for small numbers of training positives, but this difference decreases with the number of training positives. The TF *MafF* is a clear example of this, the performance difference between genomic sampling and cell line sampling is initially very large, almost 20% AUROC, but converges with more positives. In contrast, the TF *USF-1* shows a very small difference in performance between negative types (except for shuffled negatives) even for small numbers of training positives. In general, the behavior for models trained on HQ dataset does not seem to deviate from those trained with other negatives. They also exhibit similar plateauing and convergence with increasing positives.

These results reveal a complex interplay between the number of training positives, the choice of negatives, and the TF. The choice of negatives is especially important for TFs with limited training data. It should be noted that our cross-validation strategy results in only approximately 1/3 of the positives being used for training. However, for many TFs the total number of positives across all splits remains below 10 000, well within the range tested in Fig. 3. Consequently, the number of training positives is likely a limiting factor for the performance even when a different cross-validation strategy is used. Additionally, the added advantage of the HQ dataset diminishes with more data. As a result, increasing the number of positives, by for instance merging different ChIP-seq datasets for the same TF, might be an easier alternative to the construction of HQ datasets, which require matched ATAC-seq data.

### 3.6 Cross-TF performance

**Figure 4:**
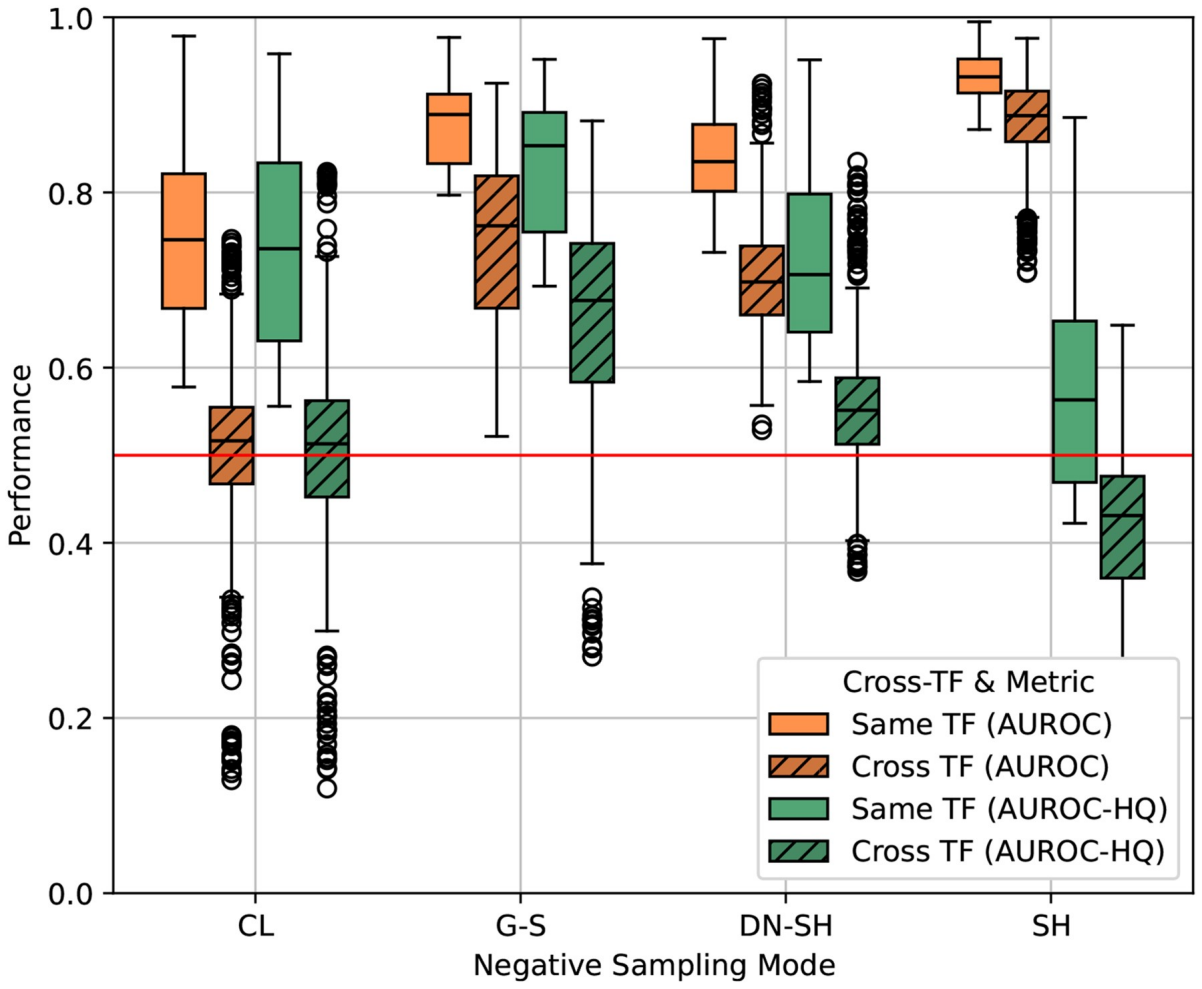
Performance of models when tested on their own training TF (*Same TF*) versus other TFs (*Other TF*). The *x*-axis shows dataset types: *SH* (shuffled), *DN-SH* (dinucleotide-shuffled), *CL* (cell line negatives), and *G-S* (genomic sampling).

The large performance gap between standard and HQ AUROC scores raises questions as to what the models are truly learning. To further investigate this, we tested some trained models on the datasets of other TFs. This was only done for the *A549* cell line. Heatmaps for models trained on shuffled, genome-sampled, and cell line sampled datasets are shown in Fig. 3, available as supplementary data at *Bioinformatics* online. An additional summarization of these results is shown in Fig. 4, where each box plot summarizes the performance of the models when tested on either their own dataset (the diagonal of the heatmaps in Fig. 3, available as supplementary data at *Bioinformatics* online) or when tested on other TFs (the off-diagonal elements of the heatmaps in Fig. 3, available as supplementary data at *Bioinformatics* online). If models are learning TF-specific binding patterns, we expect to see a diagonal pattern in Fig. 3, available as supplementary data at *Bioinformatics* online. Models should perform well on their own datasets, but should fail at predicting the binding of other TFs. Similarly, in Fig. 4, we expect the box plots to show a clear difference between the normal and cross-TF performance. For shuffled datasets, this pattern is clearly absent. Models trained on one TF performs similarly on others, indicating that they might have learned to differentiate between real and shuffled genomic sequences. This is supported by the fact that the HQ AUROC scores are very low. For the cell line sampled datasets, the situation is different. The cross-TF performance is close to random and a clear diagonal is visible on the heatmap. This is expected, as they have been specifically trained to reject the binding sites of the other TFs. Surprisingly, models trained on genome-sampled datasets also show good cross-TF performance, which in this case does seem to transfer to HQ datasets. This might indicate that there is some commonality in the binding patterns of the TFs that the model is able to capture.

These three different types of datasets show that the choice of negatives can lead to models optimized for different tasks. While shuffled datasets may be unsuitable, the choice between cell line-sampled and genome-sampled datasets depends on the use case. Cell line sampled datasets are optimized for TF-specific performance, while genome-sampled datasets are optimized for general binding of any TF. The former, the ability to predict where specific TFs will bind and the ability to discern this from the binding positions of other TFs, is the more relevant biological question. Our results indicate that the paradigm of training separate models for each TF, which is common in the literature, might not be the most appropriate for answering this question. Instead, multi-label models [which are already being explored in the literature (Zhou and Troyanskaya 2015, Quang and Xie 2016, Hiranuma *et al.* 2017)] that incorporate instance-wise performance metrics might be more suitable. It should be noted that the current training setup is not optimal for either task. If the goal is to create a general, non-specific, TF binding model, there is no need to use separate datasets for each TF. Instead, a single model could be trained on the combined dataset, as has been done in the literature (Qin and Feng 2017, Shen *et al.* 2018, Zhang *et al.* 2022b, c).

### 3.7 Cross-cell-line performance

Finally, the cross-cell-line performance of the models was also investigated. Some TFs occur in multiple cell lines, allowing us to test models trained on one cell line on the dataset of another. Figures 4 and 5, available as supplementary data at *Bioinformatics* online show the results for models trained on GM12878 and tested on both the standard as HQ datasets of other cell lines. The heatmap shows values averaged of cross validation splits. Generally, we see that the models perform similarly when tested on other cell lines. This indicates that the models are learning general binding patterns of TFs that are consistent over cell lines. However, sometimes there is a larger drop in performance. This is most pronounced in ATF3 and ZBTB33, which indicates that these models might be overfitting on cell line specific patterns. The fact that this is not a general trends points to a biological cause rather than a technical one. Interestingly, the drop in performance is different between cell lines, and generally the drop is the most pronounced when testing on A549. This indicates that this cell line might be more epigenetically different from GM12878 than the other cell lines.

## 4 Conclusion

In this work, we evaluated the impact of negative sampling strategies on the performance of deep learning-based TFBS prediction models using high-quality test datasets constructed using matching ChIP-seq and ATAC-seq data. This enabled us to validate model performance using a realistic metric. Our results show that the choice of negative sampling strongly influences model performance. Models trained on genome-sampled datasets achieved the highest performance on high-quality test sets, whereas dinucleotide shuffling, despite its widespread use, produced overoptimistic estimates and poor real-world performance. Negative sampling strategies that incorporate real genomic sequences generally yielded higher performance and lower bias. Surprisingly, a simple PWM-based approach outperformed models trained on dinucleotide-shuffled, shuffled, and even cell line-sampled datasets. Model performance was also influenced by the number of positives: more positives improved performance and reduced differences between negative sampling strategies. These findings suggest that genomic sampling with sufficient positives is a strong alternative to high-quality datasets, though not a complete substitute. We recommend validating future findings on high-quality datasets whenever feasible.

Our analysis further showed that different negative sampling strategies optimize models for different tasks. Models can be optimized for TF-specific binding or general TF binding, with the former being more biologically relevant. The current approach may be suboptimal for either objective. Future work should explore multi-label models with instance-wise performance metrics and explicitly compare them to single-TF models. Additionally, new negative sampling strategies tailored for TF-specific models could be explored. Beyond negative sampling, future research should also consider merging ChIP-seq datasets for the same TF to increase the number of positives.

## Supplementary Material

btag048_Supplementary_Data

## Data Availability

All data used in this work is publicly available from ENCODE and maxATAC (DOI: https://doi.org/10.5281/zenodo.6761768).
