# Supplementary material for: How negative sampling shapes the performance of transcription factor binding site prediction models
Source: Bioinformatics. 2026 Jan 27;42(2):btag048. doi: 10.1093/bioinformatics/btag048 (PMC12910371; doi:10.1093/bioinformatics/btag048)
Supplement: btag048_Supplementary_Data [file btag048_supplementary_data.pdf]

Supplementary Material

Table 1. Overview of the most common methods for TFBS prediction and the type of negatives they use during training.

Method	Works
Epigenetic Information	Shrikumar et al. [2017], Quang and Xie [2019], Fu et al. [2020], Zhou et al. [2021], Yang and Henao [2022], Chen et al. [2021], Cazares et al. [2022]
Base Resolution	Avsec et al. [2021], Zhang et al. [2021c,a, 2022d]
Dinucleotide Shuffled	Alipanahi et al. [2015], Blum and Kollmann [2019], Luo et al. [2019], Zhang et al. [2020b], Shen et al. [2021], Li et al. [2021], Cao et al. [2022], Wang et al. [2022], Zhang et al. [2022c], Yin et al. [2022], Yu et al. [2023], Ding et al. [2023], Ghosh et al. [2024], Wang et al. [2024a,b], Zhang et al. [2024], Wei et al. [2024], Zhang et al. [2022b]
Genomic Sampling	Qin and Feng [2017], Shen et al. [2018], Yang et al. [2019], Zhang et al. [2019b], Zhou et al. [2019], Zhang et al. [2019a, 2020a], Deng et al. [2021], Zhang et al. [2021b], Jing et al. [2022a,b], Li et al. [2022], Qi et al. [2024]
Neighborhood Sampling	Zhang et al. [2022a], Zhuang et al. [2024]
Cross-TF Sampling	Zhou and Troyanskaya [2015], Quang and Xie [2016], Hiranuma et al. [2017]

Table 1 References

B. Alipanahi, A. Delong, M. T. Weirauch, and B. J. Frey. Predicting the sequence specificities of DNA- and RNA-binding proteins by deep learning. *Nat Biotechnol*, 33(8):831–838, Aug. 2015. ISSN 1546-1696. doi: 10.1038/nbt.3300.

Ž. Avsec, M. Weilert, A. Shrikumar, S. Krueger, A. Alexandari, K. Dalal, R. Fropf, C. McAnany, J. Gagneur, A. Kundaje, and J. Zeitlinger. Base-resolution models of transcription-factor binding reveal soft motif syntax. *Nat Genet*, 53(3):354–366, Mar. 2021. ISSN 1546-1718. doi: 10.1038/s41588-021-00782-6.

C. F. Blum and M. Kollmann. Neural networks with circular filters enable data efficient inference of sequence motifs. *Bioinformatics*, 35(20): 3937–3943, Oct. 2019. ISSN 1367-4803. doi: 10.1093/bioinformatics/btz194.

L. Cao, P. Liu, J. Chen, and L. Deng. Prediction of Transcription Factor Binding Sites Using a Combined Deep Learning Approach. *Front Oncol*, 12:893520, June 2022. ISSN 2234-943X. doi: 10.3389/fonc.2022.893520.

T. A. Cazares, F. W. Rizvi, B. Iyer, X. Chen, M. Kotliar, A. T. Bejjani, J. A. Wayman, O. Donmez, B. Wronowski, S. Parameswaran, L. C. Kottyan, A. Barski, M. T. Weirauch, V. S. Prasath, and E. R. Miraldi. maxATAC: Genome-scale transcription-factor binding prediction from ATAC-seq with deep neural networks, July 2022.

C. Chen, J. Hou, X. Shi, H. Yang, J. A. Birchler, and J. Cheng. DeepGRN: Prediction of transcription factor binding site across cell-types using attention-based deep neural networks. *BMC Bioinformatics*, 22(1):38, Feb. 2021. ISSN 1471-2105. doi: 10.1186/s12859-020-03952-1.

L. Deng, H. Wu, X. Liu, and H. Liu. DeepD2V: A Novel Deep Learning-Based Framework for Predicting Transcription Factor Binding Sites from Combined DNA Sequence. *Int J Mol Sci*, 22(11):5521, May 2021. ISSN 1422-0067. doi: 10.3390/ijms22115521.

P. Ding, Y. Wang, X. Zhang, X. Gao, G. Liu, and B. Yu. DeepSTF: Predicting transcription factor binding sites by interpretable deep neural networks combining sequence and shape. *Briefings in Bioinformatics*, 24(4):bbad231, July 2023. ISSN 1477-4054. doi: 10.1093/bib/bbad231.

L. Fu, L. Zhang, E. Dollinger, Q. Peng, Q. Nie, and X. Xie. Predicting transcription factor binding in single cells through deep learning. *Science Advances*, 6(51):eaba9031, Dec. 2020. doi: 10.1126/sciadv.aba9031.

N. Ghosh, D. Santoni, I. Saha, and G. Felici. Predicting Transcription Factor Binding Sites with Deep Learning. *International Journal of Molecular Sciences*, 25(9):4990, Jan. 2024. ISSN 1422-0067. doi: 10.3390/ijms25094990.

N. Hiranuma, S. Lundberg, and S.-I. Lee. DeepATAC: A deep-learning method to predict regulatory factor binding activity from ATAC-seq signals, Aug. 2017.

F. Jing, S.-W. Zhang, and S. Zhang. Prediction of Transcription Factor Binding Sites With an Attention Augmented Convolutional Neural Network. *IEEE/ACM Transactions on Computational Biology and Bioinformatics*, 19(6):3614–3623, Nov. 2022a. ISSN 1557-9964. doi: 10.1109/TCBB.2021.3126623.

F. Jing, S.-W. Zhang, and S. Zhang. Prediction of Transcription Factor Binding Sites With an Attention Augmented Convolutional Neural Network. *IEEE/ACM Transactions on Computational Biology and Bioinformatics*, 19(6):3614–3623, Nov. 2022b. ISSN 1557-9964. doi: 10.1109/TCBB.2021.3126623.

B. Li, Z. Wang, S. Xiong, and Y. Zhang. Densely Convolutional Neural Network for Transcription Factor Binding Sites Prediction Using DNA Sequence and Histone Modification. In *2022 5th International Conference on Artificial Intelligence and Big Data (ICAIBD)*, pages 179–183, May 2022. doi: 10.1109/ICAIBD55127.2022.9820177.

J.-Y. Li, S. Jin, X.-M. Tu, Y. Ding, and G. Gao. Identifying complex motifs in massive omics data with a variable-convolutional layer in deep neural network. *Briefings in Bioinformatics*, 22(6):bbab233, Nov. 2021. ISSN 1477-4054. doi: 10.1093/bib/bbab233.

X. Luo, W. Chi, and M. Deng. Deepprune: Learning Efficient and Interpretable Convolutional Networks Through Weight Pruning for Predicting DNA-Protein Binding. *Frontiers in Genetics*, 10, 2019. ISSN 1664-8021.

T. Qi, Y. Zhou, Y. Sheng, Z. Li, Y. Yang, Q. Liu, and Q. Ge. Prediction of Transcription Factor Binding Sites on Cell-Free DNA Based on Deep Learning. *J. Chem. Inf. Model.*, 64(10):4002–4008, May 2024. ISSN 1549-9596. doi: 10.1021/acs.jcim.4c00047.

Q. Qin and J. Feng. Imputation for transcription factor binding predictions based on deep learning. *PLOS Computational Biology*, 13(2): e1005403, Feb. 2017. ISSN 1553-7358. doi: 10.1371/journal.pcbi.1005403.

D. Quang and X. Xie. DanQ: A hybrid convolutional and recurrent deep neural network for quantifying the function of DNA sequences. *Nucleic Acids Res*, 44(11):e107, June 2016. ISSN 0305-1048. doi: 10.1093/nar/gkw226.

D. Quang and X. Xie. FactorNet: A deep learning framework for predicting cell type specific transcription factor binding from nucleotide-resolution sequential data. *Methods*, 166:40–47, Aug. 2019. ISSN 1046-2023. doi: 10.1016/j.ymeth.2019.03.020.

L.-C. Shen, Y. Liu, J. Song, and D.-J. Yu. SAResNet: Self-attention residual network for predicting DNA-protein binding. *Brief Bioinform*, 22 (5):bbab101, Apr. 2021. ISSN 1467-5463. doi: 10.1093/bib/bbab101.

Z. Shen, W. Bao, and D.-S. Huang. Recurrent Neural Network for Predicting Transcription Factor Binding Sites. *Sci Rep*, 8(1):15270, Oct. 2018. ISSN 2045-2322. doi: 10.1038/s41598-018-33321-1.

A. Shrikumar, P. Greenside, and A. Kundaje. Reverse-complement parameter sharing improves deep learning models for genomics, Jan. 2017.

K. Wang, X. Zeng, J. Zhou, F. Liu, X. Luan, and X. Wang. BERT-TFBS: A novel BERT-based model for predicting transcription factor binding sites by transfer learning. *Brief Bioinform*, 25(3):bbae195, May 2024a. ISSN 1467-5463. doi: 10.1093/bib/bbae195.

W. Wang, X. Jiao, B. Sun, S. Liang, X. Wang, and Y. Zhou. DeepGenBind: A novel deep learning model for predicting transcription factor binding sites. In *2022 IEEE International Conference on Bioinformatics and Biomedicine (BIBM)*, pages 3629–3635, Dec. 2022. doi: 10.1109/BIBM55620.2022.9994984.

X. Wang, L. Qiao, P. Qu, and Q. Yang. TBCA: Prediction of transcription factor binding sites using a deep neural network with lightweight attention mechanism. *IEEE J Biomed Health Inform*, PP, Jan. 2024b. ISSN 2168-2208. doi: 10.1109/JBHI.2024.3355758.

Y. Wei, Q. Zhang, and L. Liu. Predicting transcription factor binding sites by a multi-modal representation learning method based on cross-attention network. *Applied Soft Computing*, 166:112134, Nov. 2024. ISSN 1568-4946. doi: 10.1016/j.asoc.2024.112134.

J. Yang, A. Ma, A. D. Hoppe, C. Wang, Y. Li, C. Zhang, Y. Wang, B. Liu, and Q. Ma. Prediction of regulatory motifs from human Chip-sequencing data using a deep learning framework. *Nucleic Acids Research*, 47(15):7809–7824, Sept. 2019. ISSN 0305-1048. doi: 10.1093/nar/gkz672.

T. Yang and R. Henao. TAMC: A deep-learning approach to predict motif-centric transcriptional factor binding activity based on ATAC-seq profile. *PLOS Computational Biology*, 18(9):e1009921, Sept. 2022. ISSN 1553-7358. doi: 10.1371/journal.pcbi.1009921.

Y.-H. Yin, L.-C. Shen, Y. Jiang, S. Gao, J. Song, and D.-J. Yu. Improving the prediction of DNA-protein binding by integrating multi-scale dense convolutional network with fault-tolerant coding. *Analytical Biochemistry*, 656:114878, Nov. 2022. ISSN 0003-2697. doi: 10.1016/j.ab.2022.114878.

Y. Yu, P. Ding, H. Gao, G. Liu, F. Zhang, and B. Yu. Cooperation of local features and global representations by a dual-branch network for transcription factor binding sites prediction. *Briefings in Bioinformatics*, 24(2):bbad036, Mar. 2023. ISSN 1477-4054. doi: 10.1093/bib/bbad036.

Q. Zhang, Z. Shen, and D.-S. Huang. Modeling in-vivo protein-DNA binding by combining multiple-instance learning with a hybrid deep neural network. *Sci Rep*, 9(1):8484, June 2019a. ISSN 2045-2322. doi: 10.1038/s41598-019-44966-x.

Q. Zhang, L. Zhu, and D.-S. Huang. High-Order Convolutional Neural Network Architecture for Predicting DNA-Protein Binding Sites. *IEEE/ACM Transactions on Computational Biology and Bioinformatics*, 16(04):1184–1192, July 2019b. ISSN 1545-5963. doi: 10.1109/TCBB.2018.2819660.

Q. Zhang, L. Zhu, W. Bao, and D.-S. Huang. Weakly-Supervised Convolutional Neural Network Architecture for Predicting Protein-DNA Binding. *IEEE/ACM Transactions on Computational Biology and Bioinformatics*, 17(2):679–689, Mar. 2020a. ISSN 1557-9964. doi: 10.1109/TCBB.2018.2864203.

Q. Zhang, S. Wang, Z. Chen, Y. He, Q. Liu, and D.-S. Huang. Locating transcription factor binding sites by fully convolutional neural network. *Brief Bioinform*, 22(5):bbaa435, Jan. 2021a. ISSN 1467-5463. doi: 10.1093/bib/bbaa435.

Q. Zhang, W. Yu, K. Han, A. K. Nandi, and D.-S. Huang. Multi-Scale Capsule Network for Predicting DNA-Protein Binding Sites. *IEEE/ACM Transactions on Computational Biology and Bioinformatics*, 18(5):1793–1800, Sept. 2021b. ISSN 1557-9964. doi: 10.1109/TCBB.2020.3025579.

Q. Zhang, Y. He, S. Wang, Z. Chen, Z. Guo, Z. Cui, Q. Liu, and D.-S. Huang. Base-resolution prediction of transcription factor binding signals by a deep learning framework. *PLOS Computational Biology*, 18(3):e1009941, Mar. 2022a. ISSN 1553-7358. doi: 10.1371/journal.pcbi.1009941.

S. Zhang, L. Yang, X. Wu, N. Sheng, Y. Fu, A. Ma, and Y. Wang. GraphPred: An approach to predict multiple DNA motifs from ATAC-seq data using graph neural network and coexisting probability, May 2022b.

S. Zhang, L. Yang, X. Wu, N. Sheng, Y. Fu, A. Ma, and Y. Wang. MMGraph: A multiple motif predictor based on graph neural network and coexisting probability for ATAC-seq data. *Bioinformatics*, 38(19):4636–4638, Oct. 2022c. ISSN 1367-4803. doi: 10.1093/bioinformatics/btac572.

Y. Zhang, S. Qiao, S. Ji, and Y. Li. DeepSite: Bidirectional LSTM and CNN models for predicting DNA–protein binding. *Int. J. Mach. Learn. & Cyber.*, 11(4):841–851, Apr. 2020b. ISSN 1868-808X. doi: 10.1007/s13042-019-00990-x.

Y. Zhang, Z. Wang, Y. Zeng, J. Zhou, and Q. Zou. High-resolution transcription factor binding sites prediction improved performance and interpretability by deep learning method. *Briefings in Bioinformatics*, 22(6):bbab273, Nov. 2021c. ISSN 1477-4054. doi: 10.1093/bib/bbab273.

Y. Zhang, Y. Liu, Z. Wang, M. Wang, S. Xiong, and Q. Zou. Predicting cell type-specific effects of variants on TF-DNA binding by meta-learning. In *2022 IEEE International Conference on Bioinformatics and Biomedicine (BIBM)*, pages 680–685, Dec. 2022d. doi: 10.1109/BIBM55620.2022.9995698.

Y. Zhang, Z. Wang, F. Ge, X. Wang, Y. Zhang, S. Li, Y. Guo, J. Song, and D.-J. Yu. MLSNet: A deep learning model for predicting transcription factor binding sites. *Briefings in Bioinformatics*, 25(6):bbae489, Nov. 2024. ISSN 1477-4054. doi: 10.1093/bib/bbae489.

H. Zhou, A. Shrikumar, and A. Kundaje. Towards a Better Understanding of Reverse-Complement Equivariance for Deep Learning Models in Regulatory Genomics, Feb. 2021.

J. Zhou and O. G. Troyanskaya. Predicting effects of noncoding variants with deep learning–based sequence model. *Nat Methods*, 12(10):931–934, Oct. 2015. ISSN 1548-7091. doi: 10.1038/nmeth.3547.

J. Zhou, Q. Lu, L. Gui, R. Xu, Y. Long, and H. Wang. MTTFsite: Cross-cell type TF binding site prediction by using multi-task learning. *Bioinformatics*, 35(24):5067–5077, Dec. 2019. ISSN 1367-4803. doi: 10.1093/bioinformatics/btz451.

J. Zhuang, X. Huang, S. Liu, W. Gao, R. Su, and K. Feng. MulTFBS: A Spatial-Temporal Network with Multichannels for Predicting Transcription Factor Binding Sites. *J. Chem. Inf. Model.*, 64(10):4322–4333, May 2024. ISSN 1549-9596. doi: 10.1021/acs.jcim.3c02088.

Table 2. An overview of the used cell lines and their corresponding transcription factors.

Cell Line	TFs	Count
GM12878	CTCF, YY1_(SC-281), TBP, Egr-1, Mxi1_(AF4185), SRF, MAZ_(ab85725), ELK1_(1277-1), SIX5, USF-1, SP1, RFX5_(200-401-194), ELF1_(SC-631), ATF2_(SC-81188), NF-YB, USF2, Znf143_(16618-1-AP), ZEB1_(SC-25388), Pbx3, MEF2A, TCF12, Max, STAT5A_(SC-74442), NFIC_(SC-81335), Nrf1, CEBPB_(SC-150), FOXM1_(SC-502), RXRA, ZBTB33, ETS1, ATF3, NF-YA, IKZF1_(IkN)_(UCLA), JunD	34
K562	ZBTB33, CTCF, Egr-1, MAZ_(ab85725), MafK_(ab50322), MafF_(M8194), Max, YY1_(SC-281), TBP, ATF3, JunD, RFX5_(200-401-194), SRF, ATF1_(06-325), SIX5, SP1, NF-YA, NF-YB, USF-1, Znf143_(16618-1-AP), ELF1_(SC-631), TEAD4_(SC-101184), CEBPB_(SC-150), FOSL1_(SC-183), SETDB1, ETS1, ZBTB7A_(SC-34508), NR2F2_(SC-271940), MEF2A, STAT5A_(SC-74442), Nrf1, Mxi1_(AF4185), ELK1_(1277-1), USF2, Bach1_(sc-14700)	35
HepG2	ZBTB33, USF-1, SP1, FOXA1_(SC-101058), CTCF, MafK_(ab50322), MafF_(M8194), FOSL2, YY1_(SC-281), JunD, ELF1_(SC-631), Mxi1_(AF4185), ATF3, RFX5_(200-401-194), Max, RXRA, ZBTB7A_(SC-34508), MAZ_(ab85725), TBP, TEAD4_(SC-101184), CEBPB_(SC-150), USF2, SRF, MYBL2_(SC-81192), NFIC_(SC-81335), ARID3A_(NB100-279), CEBPD_(SC-636), Nrf1, HSF1, TCF12, TCF7L2, BHLHE40	32
A549	CTCF, YY1_(SC-281), CREB1_(SC-240), Max, TCF12, FOSL2, ELF1_(SC-631), BHLHE40, ATF3, USF-1, ETS1, SIX5, ZBTB33, FOXA1_(SC-101058)	14
HEK293	CTCF, TCF7L2	2
IMR90	MafK_(ab50322)	1
PANC-1	TCF7L2	1

Table 3. GM12878 Summary Statistics.

TF	Positives	Pos Split 1	Pos Split 2	Pos Split 3	Negatives	Positives (%)	Pos in closed ATAC (%)
CTCF	40107	12222	12053	15832	664845	5.689324	9.38
YY1_(SC-281)	30998	8978	9385	12635	616168	4.789807	7.41
NFIC_(SC-81335)	29066	8488	8008	12570	633948	4.383919	7.60
ATF2_(SC-81188)	23490	6856	6427	10207	639389	3.543633	5.09
ELF1_(SC-631)	22998	6649	7101	9248	640654	3.465370	5.99
FOXM1_(SC-502)	22935	6564	6456	9915	630437	3.510251	4.18
TCF12	20437	5777	6163	8497	661469	2.997041	5.04
Znf143_(16618-1-AP)	20018	5921	6282	7815	685151	2.838752	4.19
MAZ_(ab85725)	18972	5355	5885	7732	661737	2.787094	5.10
SP1	18248	5455	5421	7372	643490	2.757587	3.38
Mxi1_(AF4185)	17747	5035	5307	7405	670145	2.579911	5.85
MEF2A	17612	5139	4865	7608	674970	2.542948	6.50
Egr-1	16324	4762	4919	6643	691119	2.307465	7.40
TBP	14890	4052	4415	6423	698863	2.086156	6.09
NF-YB	13305	4118	3834	5353	740649	1.764697	27.23
Max	12553	3531	3847	5175	704765	1.749991	4.86
Pbx3	9935	2919	3013	4003	746990	1.312547	16.22
USF-1	9779	2829	2985	3965	742276	1.300304	16.77
IKZF1_(IkN)_(UCLA)	9066	2398	2647	4021	758910	1.180506	7.54
USF2	9028	2612	2676	3740	744537	1.198039	8.54
SRF	8546	2529	2546	3471	735297	1.148898	10.82
STAT5A_(SC-74442)	7432	2063	2137	3232	743471	0.989742	13.00
CEBPB_(SC-150)	5798	1576	1663	2559	751076	0.766046	4.19
Nrf1	5689	1844	1645	2200	763361	0.739744	3.53
ELK1_(1277-1)	5584	1654	1731	2199	749807	0.739220	4.57
SIX5	4843	1613	1357	1873	755910	0.636606	4.85
ZEB1_(SC-25388)	4842	1389	1477	1976	772856	0.622607	10.64
RFX5_(200-401-194)	4340	1298	1300	1742	771496	0.559397	7.19
ETS1	4114	1146	1239	1729	761386	0.537427	5.71
JunD	2476	707	713	1056	787131	0.313574	2.34
ZBTB33	2144	595	633	916	787834	0.271400	10.40
NF-YA	1841	571	538	732	791163	0.232155	4.07
RXRA	1705	429	490	786	784445	0.216880	3.23
ATF3	1675	492	498	685	789840	0.211619	15.10

Table 4. HepG2 Summary Statistics

TF	Positives	Pos Split 1	Pos Split 2	Pos Split 3	Negatives	Positives (%)	Pos in closed ATAC (%)
MafK_(ab50322)	61944	19335	14790	27819	949785	6.122588	87.25
CTCF	48692	14192	14298	20202	946387	4.893280	9.63
FOXA1_(SC-101058)	43115	12281	11540	19294	866331	4.740798	16.05
MafF_(M8194)	37631	11368	9054	17209	970038	3.734460	79.82
JunD	32275	9304	7888	15083	962395	3.244795	52.33
SP1	25478	7260	7249	10969	889131	2.785671	4.96
FOSL2	25207	6745	7021	11441	921189	2.663473	11.43
USF-1	21893	6194	6098	9601	981070	2.182832	27.73
Mxi1_(AF4185)	20376	5780	5564	9032	932965	2.137325	2.73
CEBPB_(SC-150)	18123	5107	4677	8339	979167	1.817225	18.26
ELF1_(SC-631)	17996	5118	4968	7910	962002	1.836330	5.47
MYBL2_(SC-81192)	17921	4676	4342	8903	936161	1.878350	4.34
YY1_(SC-281)	17880	5029	4862	7989	970777	1.808514	4.69
ARID3A_(NB100-279)	17621	4881	4242	8498	955627	1.810535	7.39
RXRA	17072	4668	4664	7740	945497	1.773587	5.78
NFIC_(SC-81335)	16105	4364	3932	7809	947853	1.670716	5.90
TEAD4_(SC-101184)	15172	4054	3808	7310	969132	1.541394	6.26
TBP	13814	3857	3758	6199	982570	1.386413	3.77
MAZ_(ab85725)	12099	3395	3255	5449	994040	1.202518	1.98
Max	11866	3318	3114	5434	995126	1.178361	4.09
CEBPD_(SC-636)	11428	3058	2962	5408	991795	1.139129	4.23
ZBTB7A_(SC-34508)	9188	2548	2334	4306	1028777	0.885194	6.18
USF2	6290	1870	1666	2754	1043908	0.598935	24.26
RFX5_(200-401-194)	6014	1736	1488	2790	1041888	0.573909	14.15
SRF	5314	1457	1362	2495	1047177	0.504897	17.93
ATF3	3291	949	877	1465	1051158	0.312106	3.01
ZBTB33	2879	760	749	1370	1057995	0.271380	6.98
BHLHE40	2861	777	722	1362	1055637	0.270289	3.50
TCF7L2	2742	740	658	1344	1060352	0.257926	8.42
TCF12	2066	513	433	1120	1054893	0.195466	3.97
Nrf1	1904	632	489	783	1070967	0.177468	4.57
HSF1	1434	325	339	770	1070553	0.133770	3.91

Table 5. K562 Summary Statistics

TF	Positives	Pos Split 1	Pos Split 2	Pos Split 3	Negatives	Positives (%)	Pos in closed ATAC (%)
CTCF	43228	12716	13669	16843	881700	4.673661	9.48
JunD	40057	11212	13063	15782	783354	4.864764	11.12
Egr-1	36984	10817	11897	14270	864228	4.103807	22.12
MAZ_(ab85725)	33342	9369	11294	12679	801963	3.991596	3.85
Max	31442	8926	10378	12138	821780	3.685090	5.72
TEAD4_(SC-101184)	31038	8873	9722	12443	859811	3.484092	12.22
Znf143_(16618-1-AP)	29060	8333	9764	10963	880349	3.195482	14.19
ELF1_(SC-631)	27778	8134	8961	10683	869788	3.094814	11.71
MafF_(M8194)	25069	7416	7488	10165	952102	2.565467	58.69
CEBPB_(SC-150)	22250	6503	6835	8912	937581	2.318116	31.44
ZBTB7A_(SC-34508)	21706	6450	7183	8073	922849	2.298013	9.60
MafK_(ab50322)	19323	5606	5916	7801	957651	1.977842	51.08
USF-1	18521	5424	5926	7171	943548	1.925122	23.38
TBP	17557	5117	5507	6933	916483	1.879684	7.70
NR2F2_(SC-271940)	16680	4702	5472	6506	931221	1.759677	9.95
ATF1_(06-325)	14856	4130	4756	5970	932195	1.568659	7.96
YY1_(SC-281)	12677	3790	3982	4905	943080	1.326383	4.74
FOSL1_(SC-183)	11173	3029	3806	4338	969407	1.139428	9.59
ETS1	10727	3036	3618	4073	946734	1.120359	3.97
NF-YB	10095	3229	2937	3929	996879	1.002509	22.87
STAT5A_(SC-74442)	9813	2751	3103	3959	962833	1.008897	6.52
SP1	7204	2198	2333	2673	973080	0.734889	3.43
Mxi1_(AF4185)	6709	1895	2235	2579	982968	0.677898	5.01
SETDB1	5692	1776	1907	2009	1021602	0.554077	61.21
MEF2A	5623	1557	1892	2174	1001306	0.558431	16.77
SRF	4709	1354	1596	1759	1001384	0.468048	9.43
NF-YA	4287	1378	1243	1666	1014406	0.420833	5.83
Nrf1	4211	1348	1285	1578	1013458	0.413789	5.60
SIX5	4193	1401	1303	1489	1002256	0.416613	6.03
Bach1_(sc-14700)	3803	1067	1199	1537	1019955	0.371475	22.96
ZBTB33	3285	937	1050	1298	1022300	0.320305	18.39
USF2	3084	906	1053	1125	1022005	0.300852	11.19
ELK1_(1277-1)	2964	814	1038	1112	1014320	0.291364	5.67
RFX5_(200-401-194)	2203	621	722	860	1028415	0.213755	19.70
ATF3	1232	369	423	440	1033348	0.119082	11.69

Table 6. A549 Summary Statistics

TF	Positives	Pos Split 1	Pos Split 2	Pos Split 3	Negatives	Positives (%)	Pos in closed ATAC (%)
CTCF	45735	13342	13624	18769	398119	10.304064	14.60
FOSL2	28763	7796	8467	12500	406177	6.613096	14.44
TCF12	20906	5817	5829	9260	412768	4.820672	12.67
CREB1_(SC-240)	15907	4496	4598	6813	432986	3.543606	5.60
YY1_(SC-281)	10277	2807	2951	4519	456601	2.201217	8.89
Max	9891	2668	2801	4422	454030	2.132044	5.69
ELF1_(SC-631)	8618	2357	2480	3781	454574	1.860568	6.23
USF-1	8448	2170	2457	3821	431821	1.918827	18.47
FOXA1_(SC-101058)	7700	2180	2119	3401	483685	1.566999	56.58
ZBTB33	7157	1896	2039	3222	455956	1.545411	6.43
ATF3	6588	1631	1876	3081	459449	1.413622	5.25
ETS1	5541	1535	1503	2503	463300	1.181851	4.82
SIX5	4915	1488	1318	2109	468721	1.037717	6.08
BHLHE40	3125	850	890	1385	482898	0.642974	7.10

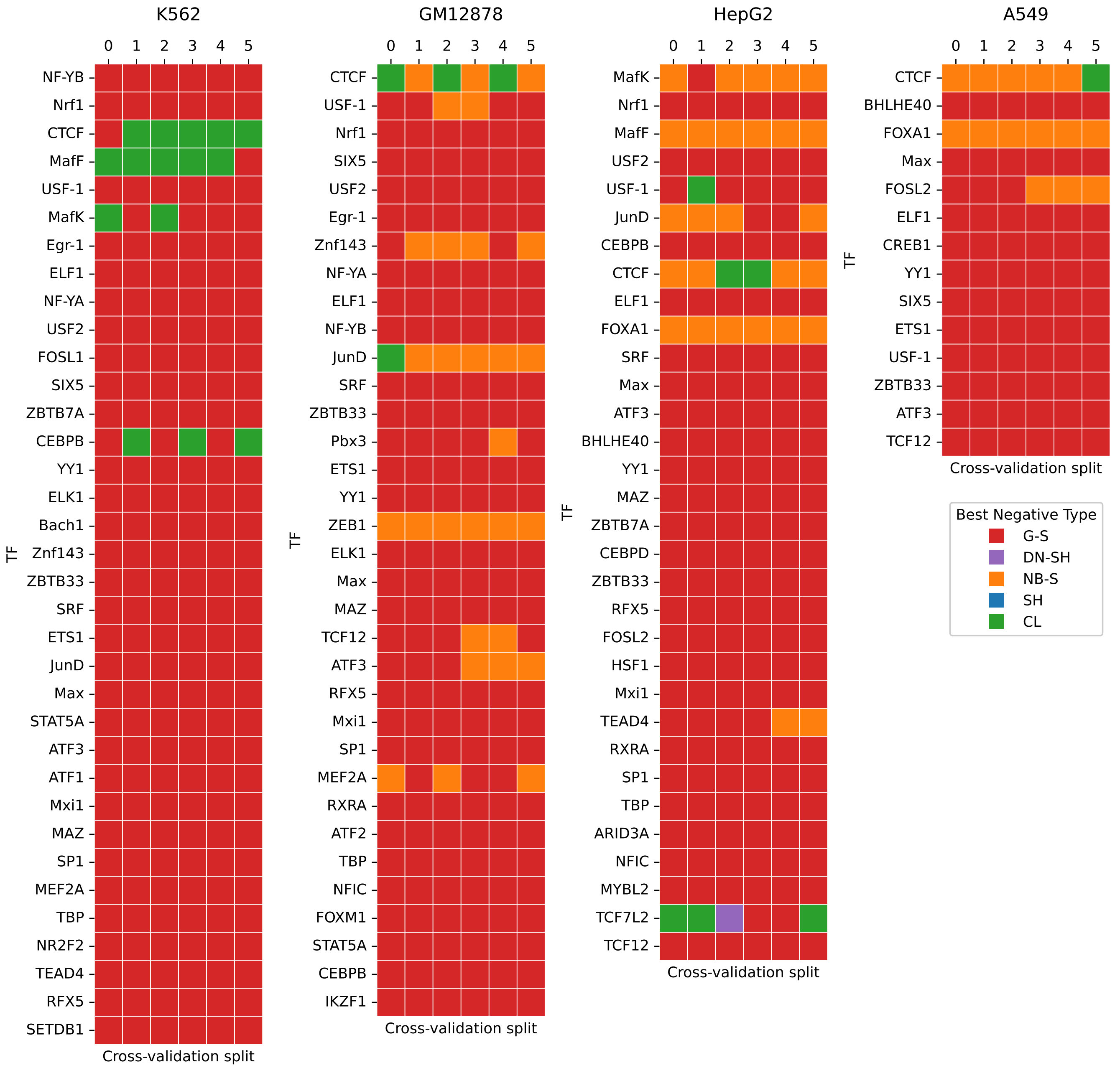

Fig. 1: Individual TF performance heatmaps, showing for each TF-cell line-cross-validation split which negative sampling type yielded the best model performance.

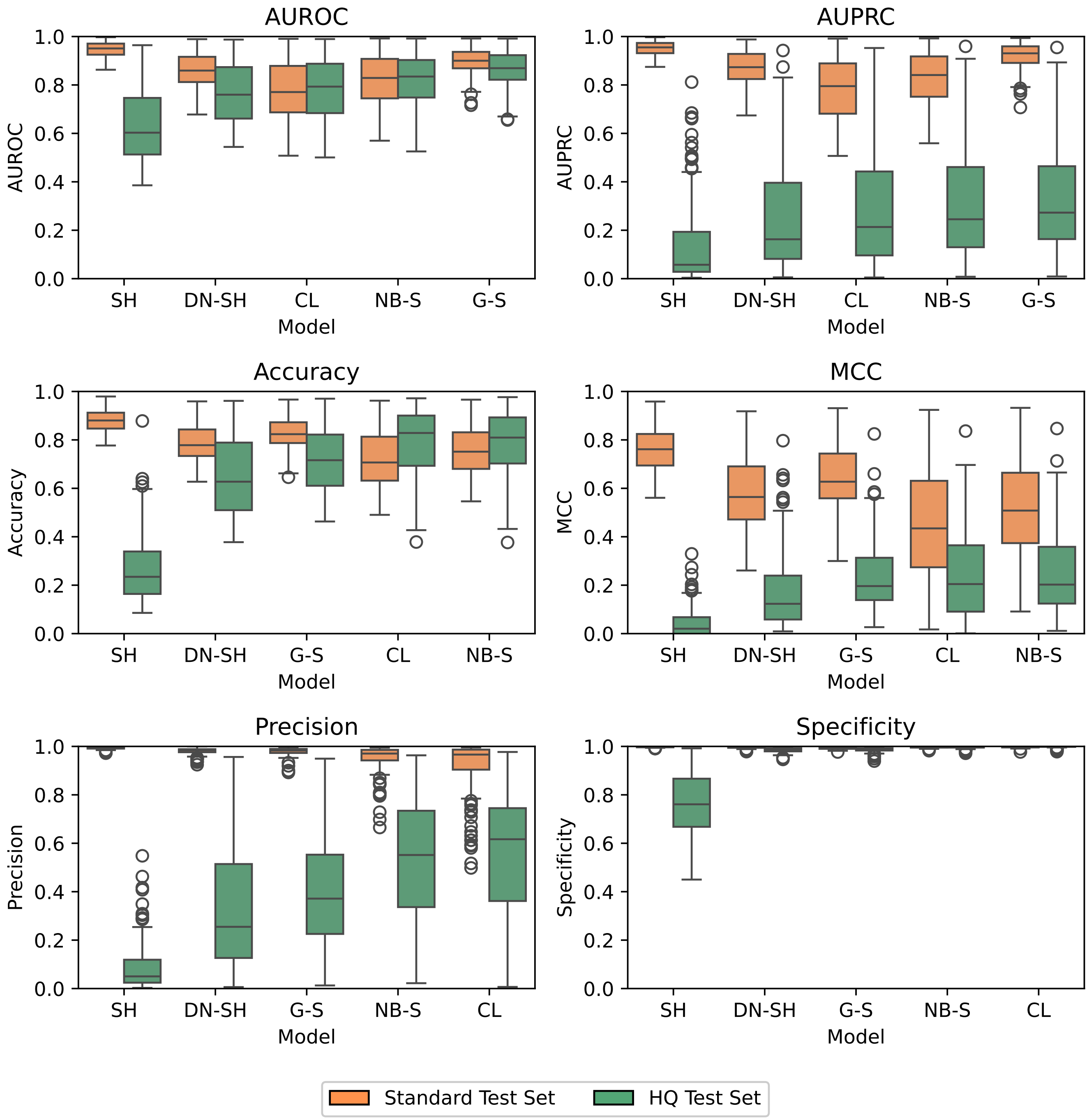

Fig. 2: Additional performance metrics AUPRC, Accuracy, MCC, Precision, and Specificity were also calculated. When metrics required thresholds, these were calculated on the validation set of the standard dataset. The results are for all cell lines and TFs combined.

Table 7. Transcription Factors and their associated JASPAR Matrix IDs

TF	Matrix_IDs
ATF2_(SC-81188)	MA1632.1 MA1632.2
ATF3	MA0605.2 MA0605.3
BHLHE40	MA0464.2 MA0464.3
Bach1_(sc-14700)	MA1633.1 MA1633.2
CEBPB_(SC-150)	MA0466.1 MA0466.2 MA0466.3 MA0466.4
CEBPD_(SC-636)	MA0836.1 MA0836.2 MA0836.3
CREB1_(SC-240)	MA0018.1 MA0018.2 MA0018.3 MA0018.4 MA0018.5
CTCF	MA0139.1 MA0139.2 MA1929.1 MA1929.2 MA1930.1 MA1930.2
ELF1_(SC-631)	MA0473.1 MA0473.2 MA0473.3 MA0473.4
ELK1_(1277-1)	MA0028.1 MA0028.2 MA0028.3
ETS1	MA0098.1 MA0098.3 MA0098.4
Egr-1	MA0162.2 MA0162.3 MA0162.4 MA0162.5
FOSL1_(SC-183)	MA0477.1 MA0477.2 MA0477.3
FOSL2	MA0478.1 MA0478.2
FOXA1_(SC-101058)	MA0148.1 MA0148.2 MA0148.3 MA0148.4 MA0148.5
FOXM1_(SC-502)	UN0802.1
GATA3_(SC-268)	MA0037.1 MA0037.2 MA0037.3
HSF1	MA0486.1 MA0486.2
IKZF1_(IkN)_(UCLA)	MA1508.1 MA1508.2
JunD	MA0491.1 MA0491.2 MA0491.3 MA0492.1 MA0492.2
MAZ_(ab85725)	MA1522.1 MA1522.2
MEF2A	MA0052.1 MA0052.2 MA0052.3 MA0052.4 MA0052.5
MYBL2_(SC-81192)	MA0777.1
MafF_(M8194)	MA0495.1 MA0495.2 MA0495.3 MA0495.4
MafK_(ab50322)	MA0496.1 MA0496.2 MA0496.3 MA0496.4
Max	MA0058.1 MA0058.2 MA0058.3 MA0058.4
Mxi1_(AF4185)	MA1108.1 MA1108.2 MA1108.3
NF-YA	MA0060.1 MA0060.2 MA0060.3 MA0060.4
NF-YB	MA0502.1 MA0502.2 MA0502.3
NFIC_(SC-81335)	MA0161.1 MA0161.2 MA0161.3 MA1527.1 MA1527.2
NR2F2_(SC-271940)	MA1111.1 MA1111.2
Nrf1	MA0506.1
Pbx3	MA1114.1 MA1114.2
RFX5_(200-401-194)	MA0510.1 MA0510.2 MA0510.3
SP1	MA0079.1 MA0079.2 MA0079.3 MA0079.4 MA0079.5
SRF	MA0083.1 MA0083.2 MA0083.3
TCF12	MA1648.1 MA1648.2
TCF7L2	MA0523.1 MA0523.2
TEAD4_(SC-101184)	MA0809.1 MA0809.2 MA0809.3
USF2	MA0526.1 MA0526.2 MA0526.3 MA0526.4 MA0526.5
YY1_(SC-281)	MA0095.1 MA0095.2
ZBTB33	MA0527.1 MA0527.2
ZBTB7A_(SC-34508)	MA0750.1 MA0750.2 MA0750.3
ZEB1_(SC-25388)	MA0103.2 MA0103.3 MA0103.4
Znf143_(16618-1-AP)	MA0088.2

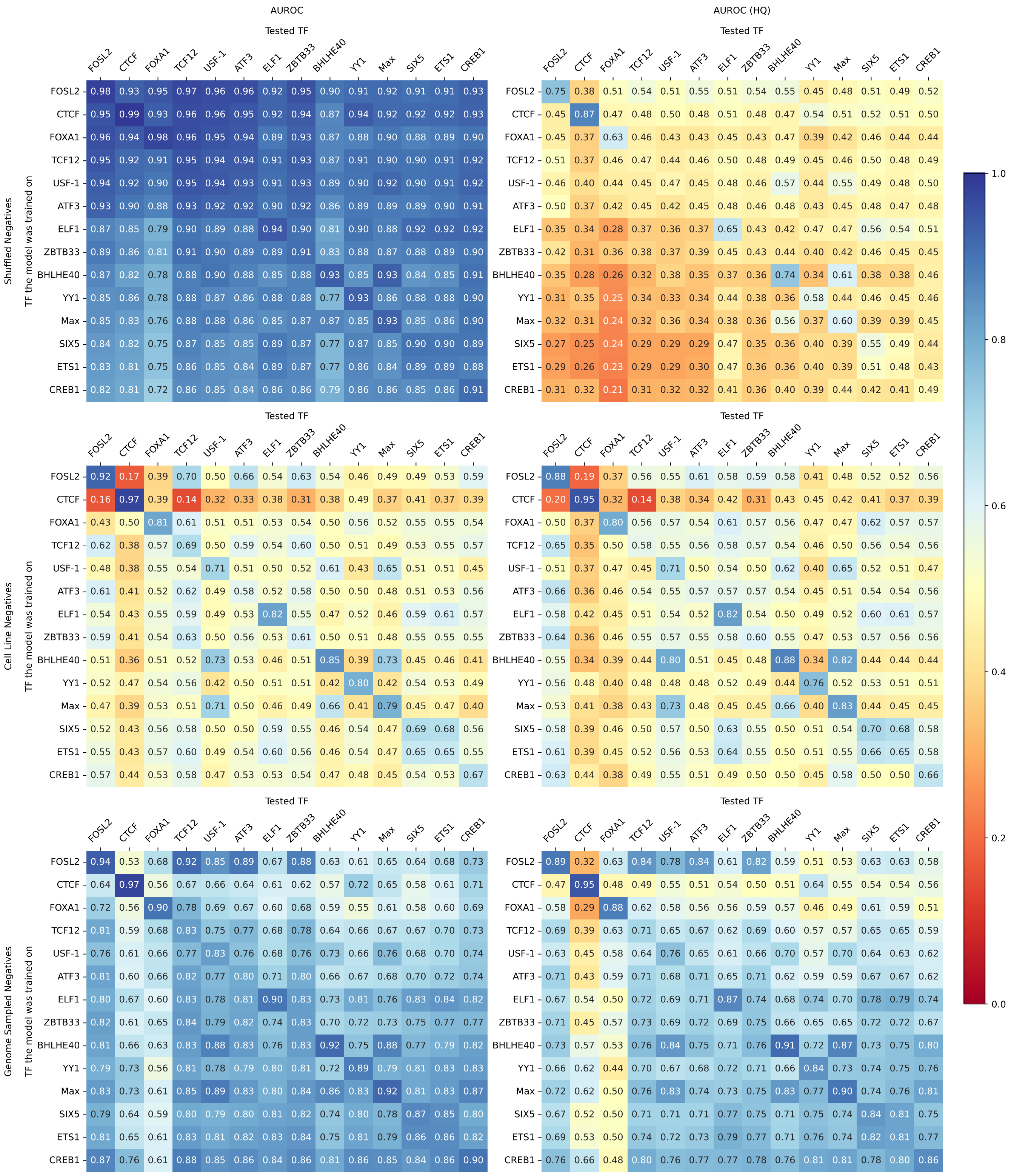

Fig. 3: Cross-TF performance heatmaps showing model performance when trained on one TF and tested on others within cell line A549.

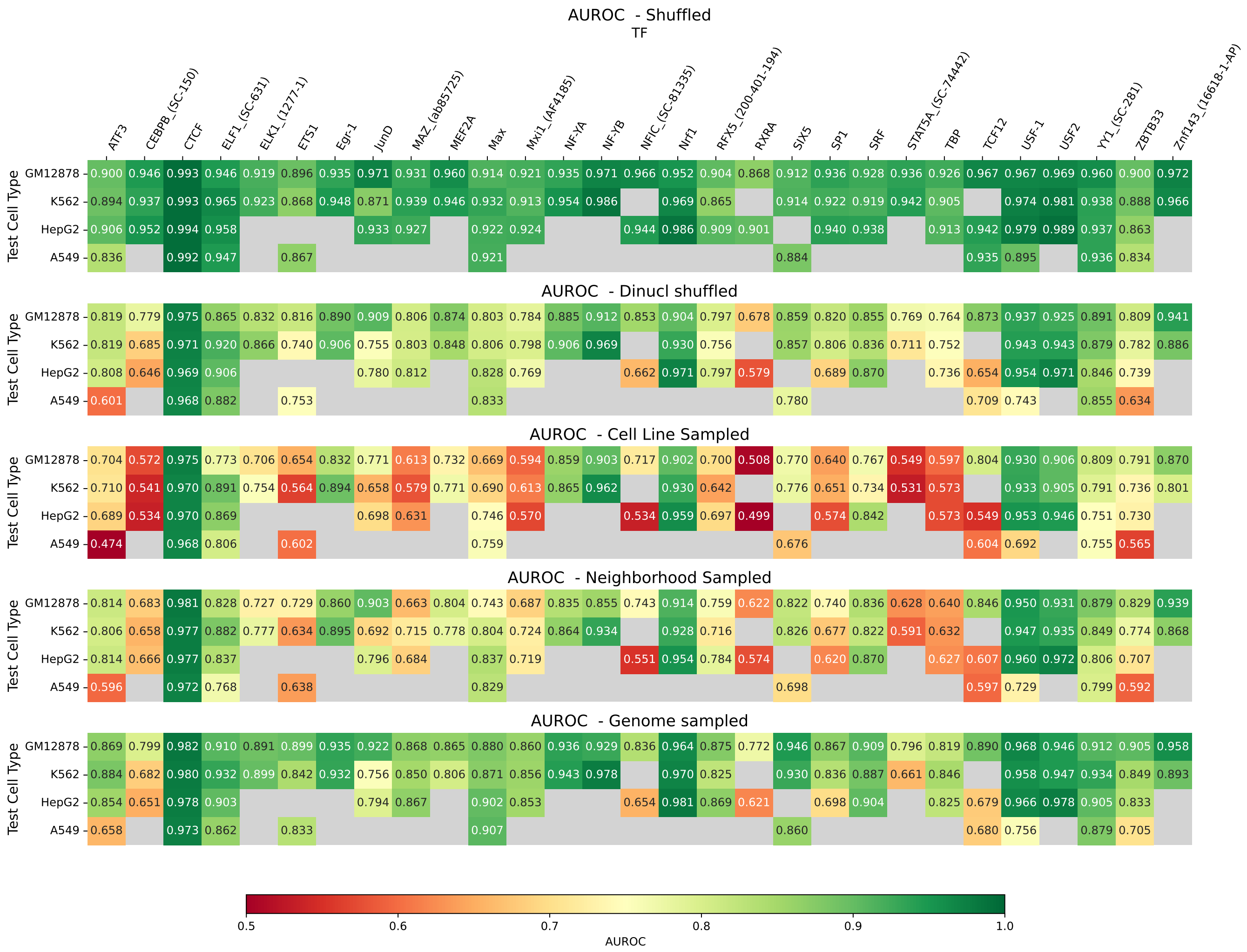

Fig. 4: Cross-cell line performance heatmaps showing AUROC performance when models are trained on GM12878 and tested on the standard test set of other cell lines. These metrics can only be calculated for TFs that are present in both the training and testing cell lines.

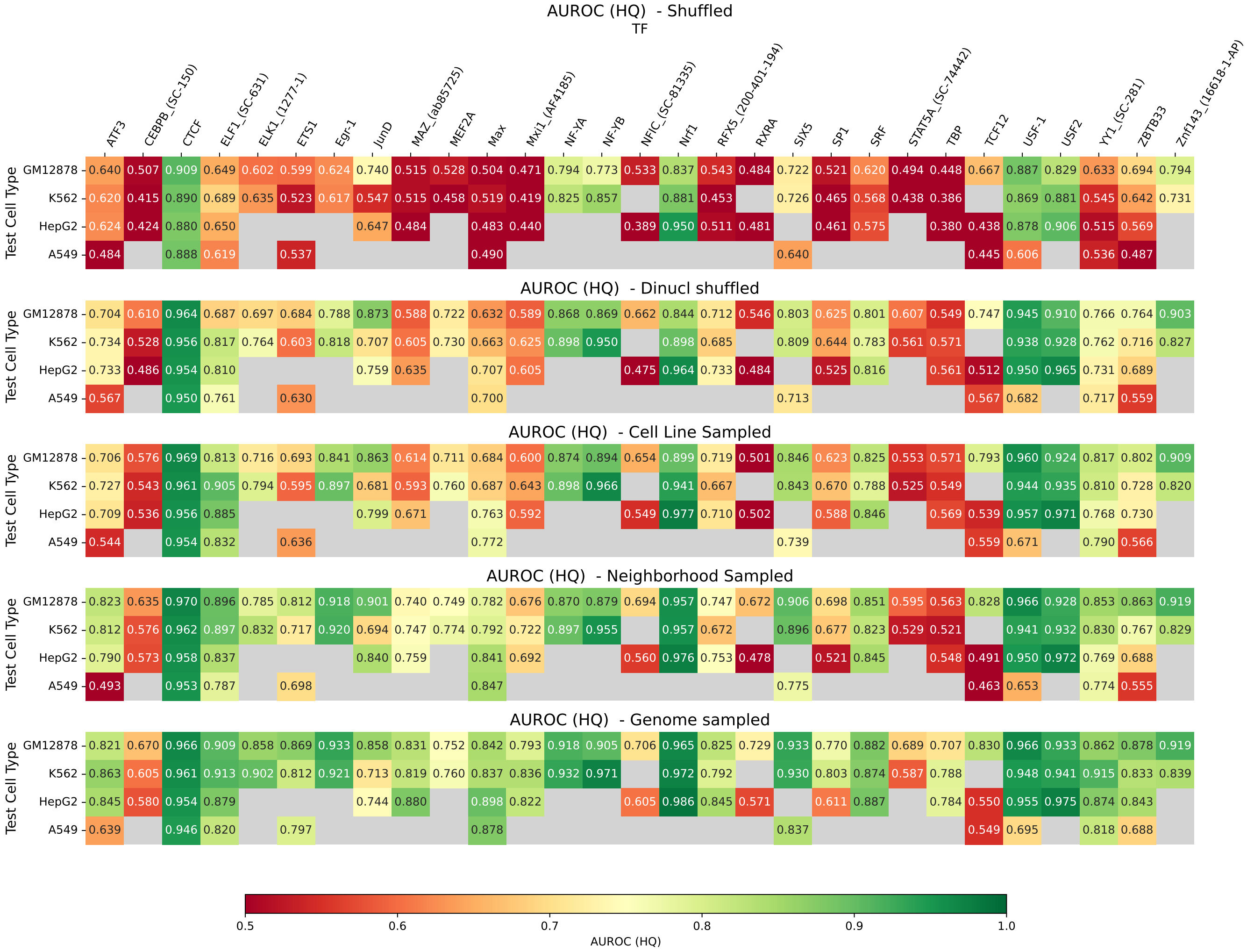

Fig. 5: Cross-cell line performance heatmaps showing AUROC performance when models are trained on GM12878 and tested on the HQ test set of other cell lines. These metrics can only be calculated for TFs that are present in both the training and testing cell lines.
